# Stigma of Dementia on Social Media During World Alzheimer’s Awareness Month: Thematic Analysis of Posts

**DOI:** 10.2196/72775

**Published:** 2025-06-02

**Authors:** Juanita-Dawne Bacsu, Jasmine Cassy Mah, Ali Akbar Jamali, Christine Conanan, Samantha Lautrup, Corinne Berger, Dylan Fiske, Sarah Fraser, Anila Virani, Florriann Fehr, Alison L Chasteen, Zahra Rahemi, Shirin Vellani, Melissa K Andrew, Allison Cammer, Katherine S McGilton, Rory Gowda-Sookochoff, Kate Nanson, Karl S Grewal, Raymond J Spiteri

**Affiliations:** 1 School of Nursing, Population Health and Aging Rural Research (PHARR) Centre, Thompson Rivers University, 805 TRU Way, Kamloops, BC, V2E2M4, Canada, 1 3062504399; 2 Department of Medicine, Dalhousie University, Halifax, NS, Canada; 3 Department of Computer Science, University of Saskatchewan, Saskatoon, SK, Canada; 4 Department of Science, Thompson Rivers University, Kamloops, BC, Canada; 5 Interdisciplinary School of Health Sciences, Faculty of Health Sciences, University of Ottawa, Ottawa, ON, Canada; 6 School of Nursing, Thompson Rivers University, Kamloops, BC, Canada; 7 Department of Psychology, University of Toronto, Toronto, ON, Canada; 8 School of Nursing, Clemson University, Clemson, SC, United States; 9 Virtual Behavioural Medicine Program, Toronto Rehabilitation Institute, University Health Network, Toronto, ON, Canada; 10 Division of Geriatric Medicine, Dalhousie University, Halifax, NS, Canada; 11 College of Pharmacy and Nurtrition, University of Saskatchewan, Saskatoon, SK, Canada; 12 Knowledge, Innovation, Talent, Everywhere (KITE) Research Institute, Toronto Rehabilitation Institute, University Health Network, Toronto, ON, Canada; 13 Department of Psychology, University of Saskatchewan, Saskatoon, SK, Canada; 14 Department of Psychology, Glenrose Rehabilitation Hospital, Edmonton, AB, Canada

**Keywords:** stigma, stereotypes, misinformation, dementia, Alzheimer disease, awareness, social media, Alzheimer's disease

## Abstract

**Background:**

Dementia-related stigma is a significant global health concern. However, public awareness and education about dementia-related stigma remain limited, especially on social media. Examining dementia-related stigma on social media is critical because it impacts how the public perceives people living with dementia. By understanding dementia-related stigma on social media, we can develop educational strategies to target false stereotypes, beliefs, and misinformation to improve the quality of life of people living with dementia.

**Objective:**

This study examines dementia-related stigma on the X platform (formerly Twitter) during World Alzheimer’s Month to identify opportunities for intervention to address dementia-related stigma.

**Methods:**

A total of 266,211 posts were scraped from X during the World Alzheimer’s Awareness Month from September 1‐30, 2022, a global advocacy campaign organized by Alzheimer’s Disease International. We used filters to exclude non-English content, duplicate posts, and reply posts with missing content. To ensure rigor and trustworthiness in the research, several measures were employed, ranging from peer debriefing sessions to documenting the research process.

**Results:**

After filtering the data, 1981 posts were examined using thematic analysis. A total of four main themes were identified including: (i) dementia stereotypes: “a burden to society”; (ii) discrimination and denied dignity: “discrimination exists in public spaces”; (iii) devaluing the lives of people with dementia: “society should legalize euthanasia”; and (iv) countering dementia-related stigma: “break down the stigma.” Although the World Alzheimer’s Awareness Month is helpful for raising awareness, more research is needed to address dementia-related stigma, stereotypes, and discrimination on social media.

**Conclusions:**

By analyzing how stigma manifests on social media, our study sheds light on the dementia education and information needed to address false beliefs, misinformation, and dementia-related stigma. The findings from our study have important implications for policymakers, health professionals, and community advocates working to design awareness campaigns to reduce dementia-related stigma on social media.

## Introduction

Dementia is a significant global health concern. Worldwide, there are an estimated 10 million new cases of dementia annually, with one new diagnosis every 3.2 seconds [1]. In 2019, the global financial cost of dementia was approximately US$ 1.3 trillion [2]. It is estimated that 57 million people live with dementia, with this number projected to triple to 153 million by 2050 with population growth and aging [3].

Although dementia is a global issue, there is limited knowledge and awareness about dementia. A survey from Alzheimer’s International [1] found that over 70% of respondents mistakenly view dementia as a normal part of aging rather than a neurodegenerative disorder. Studies show that dementia-related stigma (negative attitudes, stereotypes, and discrimination) results in social exclusion and self-internalization, and it contributes to delayed dementia diagnoses [4-6]. Moreover, dementia-related stigma can lead to health inequity [7], negative stereotypes [8], and health care discrimination [9-12].

Emerging research has started to examine dementia discourse on social media. More specifically, recent studies have reported stigmatizing language on social media [13-17]. Cheng and colleagues [17] identified stigmatization as the main theme of most of the tweets on X (formerly Twitter) regarding dementia. Another study examined dementia-related stigma on social media during the COVID-19 pandemic and identified issues of misinformation, ageism, and dementia being used as an insult for political ridicule [6]. However, no existing studies have examined the impact of global awareness campaigns on dementia-related stigma.

This study examines dementia-related stigma on X during World Alzheimer’s Month, a global advocacy campaign organized by Alzheimer’s Disease International [18]. Our study used data from X because it is a widely used platform for raising dementia awareness [19] and advocacy [20], but it has also been used to spread misinformation [21]. The X platform provides diverse insight with approximately 586 million active monthly users [22]. Moreover, emerging studies on social media awareness campaigns on health issues such as mental illness may be effective in increasing awareness, promoting help-seeking behavior [23], and reducing stigma [24]. Accordingly, X provides an effective platform to analyze dementia-related discourse during World Alzheimer’s Month to identify opportunities for intervention to reduce dementia-related stigma.

By analyzing how stigma manifests on social media, our study sheds light on the dementia education and information needed to address false beliefs, misinformation, and dementia-related stigma. Additionally, this study sheds light on the role of social media in shaping public perceptions of dementia to promote more inclusive dementia discourse. Findings from our study have important implications for policymakers, health professionals, and community advocates working to design awareness campaigns to reduce dementia-related stigma on social media.

## Methods

### Ethical Considerations

This research did not require ethics board review because it only used secondary data collected from publicly available posts, which are not classified as human subject research. The Panel on Research Ethics [25] suggests that social media research does not require research ethics board review when the study focuses solely on information that is in the public domain and the people posting the information have no reasonable belief or expectation of privacy. Similarly, the Journal of Medical Internet Research [26] notes that the analysis of large volumes of text (ie, tweets) that are publicly available on X are not typically deemed human subject research. However, we omitted the usernames and Twitter handles from our findings to help support the anonymity of the X users.

### Search Strategy

We bought a developer account for the X platform to gain access to the application programming interface (API). More specifically, the API enables developers to use data to generate material from the X platform. Using the Tweepy library [27] in Python and X’s API, we collected posts published during the annual World Alzheimer’s Awareness Month from September 1, 2022, to September 30, 2022.

The keyword search strategy was developed by examining commonly used terms and phrases on X during the World Alzheimer’s Awareness Month. We also organized team meetings to develop, review, and finalize the phrases based on their usage on the X platform. For example, we ran test searches in real time to observe the usage of specific phrases related to the World Alzheimer’s Awareness Month. This process enabled us to select terms that were being shared on X. This search strategy was based on observational patterns and trending discussions rather than using a specific tool or software.

Regarding inclusion and exclusion criteria, we included commonly used phrases and terms related to World Alzheimer’s Awareness and excluded terms that were not specific to the awareness context. More specifically, to ensure that our search captured posts related to World Alzheimer’s Month, our search phrases included the following: “World Alzheimers Month” OR “WorldAlzMonth” OR “World Alzheimer’s Month” OR “World Alzheimers Awareness Month” OR “World Alzheimer’s Awareness Month’' OR “World Alzheimer’s Day” OR “Alzheimer’s Awareness” OR “Dementia Awareness.”

We identified approximately 266,211 posts through our search. We removed 14,502 posts that were not written in English, and we excluded duplicate posts and posts that only included hashtags. We also excluded reply posts because they are often missing important background information, which left us with a total of 1,981 tweets.

### Data Analysis

Thematic analysis was used to analyze the data by following the guidelines outlined by Braun and Clarke [28]. More specifically, our thematic analysis was conducted in the following stages: (1) a thorough review of the collected data; (2) code creation and data coding; (3) extraction of the initial themes; (4) theme refinement; and (5) naming of the final themes.

Our approach to analyzing the data involved creating a codebook development team (consisting of five coauthors) to thoroughly review the posts to draft and refine the codebook. Specifically, the creation of our codebook involved the familiarization with the data through a detailed review [29] by reading and rereading the posts to develop the codebook. The codebook consisted of nine codes: (1) stopping stigma; (2) sponsorship, related to fundraising activities; (3) simple or basic posts, lacking educational content; (4) promotional and marketing content; (5) lived experiences, sharing perspectives from family, friends, or individuals with dementia; (6) fostering stigma; (7) resources, providing support information; (8) informative posts, delivering educational messages; and (9) exploitation.

Once the codebook was completed, pilot coding exercises were held to familiarize our full team of coauthors with the codebook. The first exercise included a practice coding session where the full team collaboratively coded 50 posts. Following this, more advanced exercises were undertaken, including the independent coding of 75 posts that were compared to an answer key with extensive details about why the answer was chosen. Additionally, a collective coding exercise was held where the team collaboratively coded the posts and discussed discrepancies and questions to support intercoder reliability. Once the team felt comfortable with coding, the coauthors each received approximately 208 posts to independently code and then compare the findings with a coding partner, to reconcile any differences through pair discussions and group consensus when necessary. The data analysis concluded with a full team meeting to review and finalize the naming of each theme.

### Rigor and Trustworthiness

In this study, trustworthiness and rigor were established by adhering to Lincoln and Guba’s [30] guidelines to ensure credibility, dependability, and confirmability. For example, the coding process included coding by a multidisciplinary team (with expertise in nursing, geriatrics, psychology, computer science, and biology), iterative refinement of the codebook, and independent and group coding exercises. This meticulous approach ensured that various perspectives were considered, enhancing the reliability and validity of the data analysis [28,29].

To further achieve credibility, the study employed methods such as peer debriefing, facilitated through coding exercises [31], and triangulation, using the expertise of our research team [32]. For example, our approach included partnering less experienced coders with senior coders to provide coding mentorship to further enhance intercoder reliability [33]. Dependability and confirmability were supported by developing a thorough audit trail to outline our research process (fieldnotes, codebook, and thematic map) to ensure that the study’s methodology is replicable [34].

Regular team meetings during the coding and theme development processes enhanced the study’s rigor. These sessions were foundational in addressing potential discrepancies to enhance clarity in our interpretation of the data [32]. For example, post examples were added to the codebook to enhance the clarity of the codes. Finally, providing rich descriptions of the study’s themes and supporting them with direct quotes helped to support the validity of our analysis and contribute to the study’s trustworthiness.

## Results

### Analysis Results

**Figure 1.**
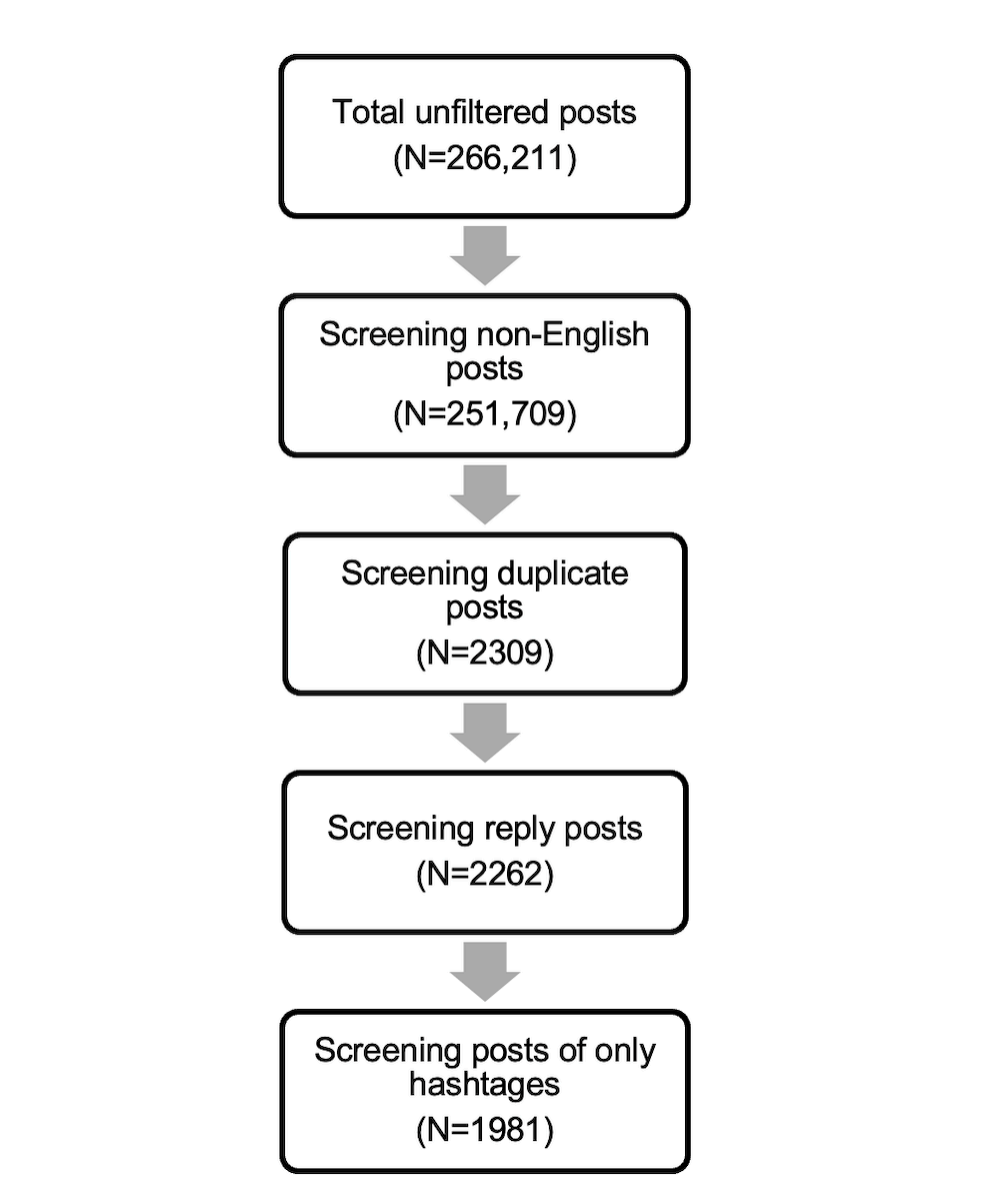
Data screening process.

The data screening process used to filter the posts is illustrated in Figure 1. Based on our thematic analysis, four main themes were identified including (1) dementia stereotypes; (2) discrimination and denied dignity; (3) devaluing the lives of people with dementia; and (4) countering dementia-related stigma.

### Theme 1: Dementia Stereotypes: “A Burden to Society”

Stereotype propagation of dementia was evident in many of the posts. Ironically, many of these posts may have been intended to be supportive but used negative language to fuel stereotypes. These posts often described people living with dementia as a burden to the economy and the health care system. These burden-laden posts are presented below:

More than 6 million Americans are living with Alzheimer’s. This #WorldAlzheimersMonth, learn about the burden of Alzheimer’s and dementia on individuals, caregivers, government, and the nation’s health care system…

It’s #WorldAlzheimersMonth. Alzheimers is not only a devastating disease, but a costly one. In 2020, total #healthcarecosts for Alzheimers treatment were estimated at $305 billion.

Posts also referred to people with dementia as “patients” rather than people. Many of these posts described people with dementia as “suffering” from the disease. However, this type of post contributes to negative stereotypes that depict all people living with dementia as suffering patients in the end-of-life stage, which further fuels the burden rhetoric.

On the eve of #WorldAlzheimersDay, which falls on September 21 every year, we look at how caregivers can support patients suffering from the condition.

…Dementia can change patients’ behavior and swing their moods, which builds up stress among others while caring for their dear parents or spouse…

It is important to recognize that people living with dementia are people who contribute to society rather than a burden as portrayed in the stereotypes.

### Theme 2: Discrimination and Denied Dignity: “Discrimination Exists in Public Spaces”

A predominant theme described discrimination and denied dignity as experienced by people living with dementia. More specifically, family members of people living with dementia shared stories of disparate treatment and public discrimination. The issues of discrimination and denied dignity are demonstrated in the following posts:

It’s Dementia Awareness week and my Mum has Alzheimer’s Dementia. She lives with us so our family sees first hand how hard and unfair this is but also how discrimination exists in public places. We are also making sure everyday counts…

Our family member lost her battle to Vascular dementia. Let’s together learn how best to support them. Stories about locking, tying them up, must stop. They need our understanding, support & love. We miss her already, very much #dementia #dementiaawareness #vasculardementia

Posts also described how stigma reduces the dignity and quality of life of people living with dementia. For example, posts highlighted that stigma is often a reality faced by people living with dementia. Stigma was often described as one of the greatest challenges impacting people living with dementia. These posts demonstrate stigma and deny dignity:

A wonderful quote from our friend... Dementia does not rob someone of their dignity. It’s our reaction to them that does. #caregiving #caregivers #caregiver #homecare #health #heathcare #respite

#Dementia is one of the biggest challenges we face, and stigma is one of the main barriers for people w/ dementia to live their lives fully with dignity…

### Theme 3: Devaluing the Lives of People With Dementia: “Society Should Legalize Euthanasia”

Another theme was devaluing the lives of people living with dementia. These tweets were typically in the form of false assumptions, patronizing sentiments, and spreading detrimental information. For example, these posts often described people with dementia as incapable of living meaningful and high-quality lives. Some posts even argued that people living with dementia were better off ending their lives than continuing to live. The following posts highlight these devaluing sentiments:

September is Alzheimer’s awareness month. I don’t wish it on anyone, and I think society should legalise euthanasia for those who don’t want to be stripped of their dignity and forced to sink into deep and upsetting confusion until their death.

Yesterday she died. It was a painful year, she was suffering so I’m glad it ended…

There was a strong focus on detailing the negative aspects of the disease. Posts described issues of people with dementia slowly slipping away and physical deterioration, rather than sharing narratives of living well with dementia.

Those who care for relatives with dementia often describe how a loved one slowly slips away, physically intact but unrecognisable bit by bit by bit…

World Alzheimer’s day Alzheimer’s is a devastating disease. It was painful to watch my person deteriorate. #mentalhealthawareness #ptsd #psychosis #mentalhealthmatters #psychology #psychiatrist

However, these posts can be harmful because they may contribute to self-stigma among people living with dementia as well as societal myths that all people with dementia are unable to live meaningful lives.

### Theme 4: Countering Dementia-Related Stigma: “Break Down the Stigma”

During World Alzheimer’s Awareness Month, many posts called for unity to dispel the stigma surrounding dementia. This theme often called for advocacy and actions to address stigmatization. For example, these posts encouraged readers to promote dementia awareness and education and “join the fight” to challenge the stigma of dementia.

#WorldAlzheimersDay is on September 21st—a time when people unite to raise awareness and attempt to break down the stigma of all forms of #dementia We can also involve children in age-appropriate caregiving…

This #WorldAlzMonth, we aim to raise awareness of and challenge the stigma around Alzheimer’s disease and other dementias.

September is #WorldAlzheimersMonth! People unite from around the world to raise awareness and challenge the stigma of Alzheimer’s disease & dementia.

#WorldAlzheimersMonth is dedicated to raising awareness and challenging the stigma surrounding dementia - join the fight today!

Other advocacy posts highlighted the need to respect people living with dementia. Some of these posts also made reference to recognizing the personhood and the human rights of people living with dementia.

…Respect our right to freedom, the right to have a say, the right to make a choice in matters related to us. #NothingAboutUsWithoutUs #KnowDementia #TogetherWeCanDoMore...

World Alzheimer’s Day - Dementia Experts Call for Care to be Recognised as a Human Right…

Dementia awareness includes understanding that changes in cognitive function or processing data does not mean a person is less intelligent, or less sensitive, or less human. #Respect still matters.

## Discussion

### Principal Findings and Comparison With Previous Works

Our study analyzed dementia-related stigma on X during World Alzheimer’s Month to identify opportunities for intervention to address the stigma of dementia. Examining the stigma of dementia is critical to understanding how stigma manifests on social media, especially during global awareness campaigns. Based on our thematic analysis, four main themes were identified, ranging from dementia stereotypes to devaluing the lives of people with dementia.

Findings from our study suggest that negative stereotypes and false information about dementia continue to persist during the global awareness campaign. For example, our study identified harmful stereotypes that depicted all people with dementia as suffering and being a burden on society. Moreover, posts often homogenize people with dementia as being incapable of living meaningful lives. There was also a strong focus on emphasizing the end-of-life stage, with some posts even suggesting that people with dementia were better off dead. Our findings are consistent with COVID-19 literature on dementia-related stigma, which reported that social media fuels misinformation, assumptions, and discrimination towards people with dementia [6]. However, dementia stereotypes and false information are harmful because they can lead to devaluing the lives of people with dementia, health inequities, and discrimination within the health care system [7,11,12].

An emerging body of literature indicates that people with dementia often self-internalize stigma and are highly concerned about being a burden on society [35-37]. Literature suggests that this concern of being a burden, combined with fears of suffering and loss of autonomy, often fuels the requests for physician-assisted suicide [36-40]. This literature on physician-assisted suicide is especially troubling when compared to our study’s findings that shed light on the dissemination of dementia-related stigma on social media in terms of associating dementia diagnosis with the end-of-life and loss of meaning and purpose of life. However, it is important to note that dementia impacts everyone differently, and not everyone’s dementia journey will be the same [41]. Accordingly, more dementia education and awareness strategies are needed to combat false information, stereotypes, and dementia-related stigma on social media.

It is important to note that organizations, such as the Alzheimer’s Association [42] and the Alzheimer Society of Canada [43], are working to address dementia-related stigma by providing accurate information. More specifically, the Alzheimer Society of Canada provides tips to reduce dementia-related stigma, ranging from using person-centered language to avoiding assumptions about people with dementia [44]. They also provide educational resources on dementia, including information on dementia screening, diagnosis, treatment, and living well with dementia [45].

### Limitations

Although our study was conducted in a rigorous manner, this research is not without limitations. More specifically, X users’ sociodemographics may not represent the general population. For example, people with lower socioeconomic status may not have access to computers or technology to access the X platform. Moreover, our study only included English-language posts, which reduces our ability to make any generalizations about dementia-related stigma across different cultures. Consequently, additional research is needed to examine the roles of sociodemographic status and culture on dementia-related stigma.

Another weakness is that using data from X may contribute to biases related to the platform’s algorithms. For example, recent literature suggests that X prioritizes and amplifies posts with higher engagement, which may increase the visibility of more sensational and low-credibility content [46]. Additionally, our study included data from all users, including nonverified accounts (possibly including chatbots and trolls), which may further contribute to bias and low-credibility discourse. However, Zhao and colleagues [47] note that there is a paucity of studies examining biases related to social media research. Accordingly, further research is needed to address the potential impact of biases related to low-credibility content and algorithms on social media research.

An additional limitation of our research may include the restricted timeframe. For example, our study focused on tweets from September 1, 2022, to September 30, 2022, to coincide with the World Alzheimer’s Awareness Month. However, this limited timeframe may not fully capture discourse on dementia-related stigma given the extensive awareness raising efforts. Accordingly, further research is needed to examine dementia-related stigma outside of World Alzheimer’s Month.

### Conclusion

Our study examined dementia-related stigma on X during the World Alzheimer’s Awareness Month. Our analysis of the data identified four main themes, ranging from dementia stereotypes to challenging stigma of dementia. Examining dementia-related stigma is essential to understanding how stigma manifests and persists on social media, especially during global awareness campaigns. By analyzing how stigma manifests on social media, our study sheds light on the dementia information and education needed to combat false beliefs, misinformation, and dementia-related stigma. Findings from our study have important implications for policymakers, health professionals, and community advocates working to design awareness campaigns to reduce dementia-related stigma on social media.

## Abbreviations

API: application programming interface
